# Unpacking the Black Box of Translation: A framework for infusing spatial thinking into curricula

**DOI:** 10.1186/s41235-020-00222-9

**Published:** 2020-06-25

**Authors:** Kristin M. Gagnier, Kelly R. Fisher

**Affiliations:** Science of Learning Institute Johns Hopkins University, 3400 N. Charles Street, Baltimore, MD 21218 USA

**Keywords:** Spatial thinking, Science education, Research-to-practice, Knowledge translation, Knowledge utilization

## Abstract

**Background:**

Spatial thinking skills are strongly correlated with achievement in Science, Technology, Engineering, and Mathematics (STEM) fields and emerging research suggests that interventions aimed at building students’ skills will likely yield measurable impacts on learning across K-12 settings. The importance of spatial thinking in science has received increased attention in academic discussions; however, the intentional practice of teaching spatial thinking skills is still largely absent from K-12 education. The translation of science into educational practice is challenging for a variety of reasons, including the difficulty “translating” research findings into practical applications and limited resources to support its development, implementation, and evaluation. Given these obstacles, one may ask “can spatial thinking be brought to the classroom?” In this paper, we argue that in order to effectively move research into the classroom, we must first systematically explore *how* spatial thinking can be translated into practice.

**Approach:**

We present a use-inspired, integrative framework that draws upon planned action and translation science theories, as well as research from cognitive, developmental, educational, and implementation sciences, to guide the infusion of spatial thinking into science curricula. In the *Knowledge Translation Framework* (KTF), translation is conceived as a multistage process, proceeding through seven stages: (1) the identification of relevant disciplinary and contextual knowledge, (2) the synthesis and translation of knowledge into guidelines to support the infusion of knowledge into the curriculum, (3) the development of tools to support curriculum development, implementation, and track the translation process, (4) the iterative development and refinement of the spatially-enhanced curriculum, (5) the creation of an analysis plan to evaluate the impact of the spatial enhancements and other contextual features on learning, (6) the development and implementation of an intervention plan, and (7) the evaluation of the intervention.

**Conclusion:**

The KTF is a use-inspired, integrative framework that unpacks the translation process and offers practical guidance on how a team may synthesize scientific and contextual knowledge, infuse it into a curriculum, and evaluate its impact in ways that will yield scientific understanding *and* practical knowledge. We also provide illustrative examples of how this approach was used to spatially enhance an elementary science curriculum.

## Significance

Some of the most transformative insights in Science, Technology, Engineering, and Mathematics (STEM) were driven by feats in spatial thinking – mental skills that enable us to reason about space. Rosalind Franklin, for instance, inferred the double-helical structure of DNA from looking at a two-dimensional x-ray and imagining what it would look like in three-dimensional space. In everyday classrooms, spatial thinking also underlies students’ STEM learning, as seen when students learn to predict the phases of the moon by visualizing how the position of the sun, moon, and earth changes the proportion of the moon that is illuminated. These examples are supported by robust research demonstrating that spatial skills are strongly correlated with STEM achievement and that building spatial skills yields measurable impacts on learning. Yet, the intentional practice of teaching spatial thinking in K-12 education is largely absent because of challenges in translating research into practice. In this paper, we provide a use-inspired framework to guide scientists and educators through this translation process and provide an illustrative example of how this approach was used to develop a spatially-enhanced science curriculum. We argue that such a framework provides a roadmap for how to translate research into education and yields critical knowledge regarding what spatial approaches are most effective in supporting learning. This type of framework will create and support stronger translational bridges between spatial thinking and educational practice that will mutually benefit science and society.

## Background

Over the last decade, scientists have increasingly advocated for the incorporation of spatial thinking into science, technology, engineering, and mathematics classrooms (STEM) (National Research Council, 2006; Newcombe, 2010, 2013, 2017; Newcombe, 2010; Verdine, Golinkoff, Hirsh-Pasek, & Newcombe, 2014a). Numerous high-quality studies indicate that spatial thinking is a critical component to students’ interest and success in STEM disciplines (Shea, Lubinski, & Benbow, 2001; Wai, Lubinski, & Benbow, 2009). Intervention studies have found that training spatial skills, compared to other forms of training, leads to improvement in a variety of STEM outcomes in a variety of age ranges (e.g., Cheng & Mix, 2014; Lowrie, Logan, & Ramful, 2017; Miller & Halpern, 2013; Sanchez, 2012; Small & Morton, 1983), including physics (Miller & Halpern, 2013), engineering (Hsi, Linn, & Bell, 1997; Sorby, 2009), calculus (Sorby, Casey, Veurink, & Dulaney, 2013), chemistry (Small & Morton, 1983), elementary and middle school math (Cheng & Mix, 2014; Lowrie et al., 2017), and geoscience (Sanchez, 2012).

Despite the robust nature of this body of work, spatial thinking is largely absent from K-12 curricula (Newcombe, 2010).

### The research-practice gap

Given the strong evidence linking spatial thinking skills and STEM success, a key question for researchers and educators naturally arises – *can* spatial thinking research findings be translated into classroom practice? Concerns about the utilization of research in educational practice have endured for over a century and continue to persist today (e.g., Dewey, 1904; Farley-Ripple, May, Karpyn, Tilley, & McDonough, 2018; Stokes, 1997; Thorndike, 1910). Such concerns have prompted explorations into the barriers contributing to the research-practice gap, with a historical focus on issues of dissemination and knowledge utilization (e.g., Bauer & Fischer, 2007; Broekkamp & Van Hout-Wolters, 2007; Farley-Ripple et al., 2018; Landry, Amara, & Lamari, 2001; Macoubrie & Harrison, 2013; McIntyre, 2005; Korthagen, 2007; Stark & Mandl, 2007; Wilson et al., 2010). Some commonly identified dissemination barriers include educators’ limited access to, and comprehensibility of, research, the difficulty of infusing such knowledge into pedagogical and curricular practices (i.e., what teachers say and do in the classroom, what activities students engage in during learning), and the limited resources and infrastructure to support the development, implementation, and evaluation of evidence-informed practices within localized contexts (e.g., Glasgow & Emmons, 2007; Metz & Bartley, 2012; Vanderlinde & van Braak, 2010).

To help address these concerns, scientists have begun disseminating their research directly to educators, school leaders, practitioners, and other audiences through a variety of public engagement and popular press activities. Indeed, in the area of spatial thinking, we have seen a growth of books, articles, press interviews, policy reports, and presentations aimed at promoting the use of research on spatial thinking in K-16 education over the last 10 years (e.g., Hegarty, 2014; Gagnier & Fisher, 2017; Newcombe, 2010, 2013, 2016, 2017; NRC, 2006; Stieff & Uttal, 2015; Uttal & Cohen, 2012; Verdine et al., 2014b; Wai & Uttal, 2018).

Despite these efforts, there is growing recognition that dissemination activities alone are insufficient to support the *translation* of research into practical applications. As Korthagen (2007) noted:

“To date, attention is more focused on how research outcomes can be better linked to practice. Probably, this has to do with the fact that all the attempts at enhancing the dissemination of research results have not led to a clear, successful, and generally accepted approach to bridging the research-practice divide and that, despite all these attempts, the gap between research and practice seems to have increased rather than diminished ….” (p. 303)

Approaches aimed at bridging the research-practice gap are often conceptualized as a one-way communication street, in which scientists “translate” their research directly to non-scientific audiences utilizing more effective dissemination strategies (e.g., clear, concise, non-jargon language, personally meaningful examples; American Association for the Advancement of Science, 2020). While effective science communication likely increases awareness, understanding, and some informal application, it does not have the necessary supports to aid *systematic* translation of research into explicit classroom practices that could result in effective uptake (e.g., Desimone et al., 2001; Graham et al., 2006; Moir, 2018). Translation, at its core, is inherently more complex and involves the transformation of scientific findings from their original research contexts (e.g., simple lessons in artificial environments, utilization of 1:1 instruction, nuanced assessment procedures, homogeneous research samples) to complex educational settings that vary in teacher expertise, student needs, resource availability, and infrastructure supports (e.g., available class time, materials, effective professional development opportunities, dedicated curriculum developers, supportive leadership). Thus, translation requires more than effective communication; it requires the synthesis of strong scientific content knowledge and a deep understanding of the educational context.

Moreover, research yields few results that can meaningfully guide the translation process (Broekkamp & Van Hout-Wolters, 2007). Research often places more emphasis on understanding the mechanisms underlying learning or the broad impact of an intervention on students’ learning outcomes (e.g., does the intervention have greater impact than the control or “business-as-usual” condition?). Few studies explore the features of learning experiences that would be useful in identifying “what works” and “for whom does it work.” For example, a number of studies have shown that gesture supports performance on mathematical equivalence problems (Broaders, Cook, Mitchell, & Goldin-Meadow, 2007; Novack, Congdon, Hemani-Lopez, & Goldin-Meadow, 2014; Singer & Goldin-Meadow, 2005). Yet the fundamental qualities that make the gesture effective (e.g., features of the hand movement, minimum number of times gesture must be used in a full 60-min math lesson) and the degree to which the gestures can be used to support students’ learning during other problems in math or other subjects (e.g., science), is unknown.

To address these issues, new perspectives arising from medicine, education, and public health emphasize the need for more use-inspired approaches in the generation, synthesis, translation, and application of knowledge (e.g., Bauer & Fischer, 2007; Broekkamp & van Hout-Wolters, 2007; Farley-Ripple et al., 2018; Glasgow & Emmons, 2007; Grzywacz & Allen, 2017; Stark & Mandl, 2007; Trochim, Kane, & Graham, 2011; Wandersman et al., 2008).

For instance, Graham et al. (2006) developed the Knowledge-to-Action Framework (KTA) to describe the process by which knowledge moves from creation into application in the health professions. KTA includes two overarching phases – the knowledge creation phase and the action phase. During the knowledge creation phase, knowledge is generated via initial inquiry (e.g., individual primary studies), which is then aggregated, synthesized, and distilled through systematic review processes into relevant knowledge. This knowledge is then translated into tools that are designed to provide explicit recommendations to meet the users’ information needs and influence what they do (e.g., health practice guidelines, decision aids, and rules of application). During the action cycle, the knowledge is reviewed, translated into interventions to meet the needs of the localized context, implemented, and evaluated.

Similarly, translational science frameworks in medicine describe the process of moving knowledge from the “bench-to-the-bedside,” in which basic animal research is translated into clinical research, then synthesized and translated into practice-based research and applications, and, ultimately, the diffusion and uptake of practices on a large scale (Trochim et al., 2011). These new approaches acknowledge the importance of bidirectional communication from across scientific and practitioner spheres in the production, synthesis, and utilization of the knowledge throughout the translation process; however, such models are meant to serve as general guides and do not delineate *how* research is translated at each stage of the process.

### Unpacking the Black Box with the Knowledge Translation Framework

To effectively bring spatial thinking into the classroom, we must first systematically explore *how* spatial thinking can be translated into education and examine its impact. Multiple issues underlie our translation deficit at the outset. First, there is a stark absence of frameworks in cognitive science to guide the translation process. The translation of scientific knowledge into real-world educational applications is, by its very nature, an interdisciplinary endeavor. A single scientific discipline alone does not contain the critical knowledge and methodological approaches necessary to effectively translate a scientific finding into relevant educational practices, programming, and infrastructure supports that work within a complex environment. Rather, it requires synthesis of scientific and professional knowledge and corresponding methods into a larger, intergrative framework. Yet it remains unclear what types of knoweldge should be utilized, how such knoweldge can be synthesized, who should have a seat at the translation table, and what role they play in the translation process (e.g, scientists, curriculum developers, professional development specialists, educational leaders).  

In this paper, we present the Knowledge Translation Framework (KTF), a use-inspired, integrative framework that draws upon research from cognitive, developmental, educational, and implementation sciences to guide the initial infusion of spatial thinking into a curricular intervention. The KTF also builds upon planned action and translational science frameworks (Graham et al., 2006) by unpacking the translation process by offering practical guidance on *how* a team may synthesize scientific and contextual knowledge, infuse it into a curriculum, and evaluate its initial impact in ways that will yield scientific understanding *and* practical knowledge.

**Fig. 1:**
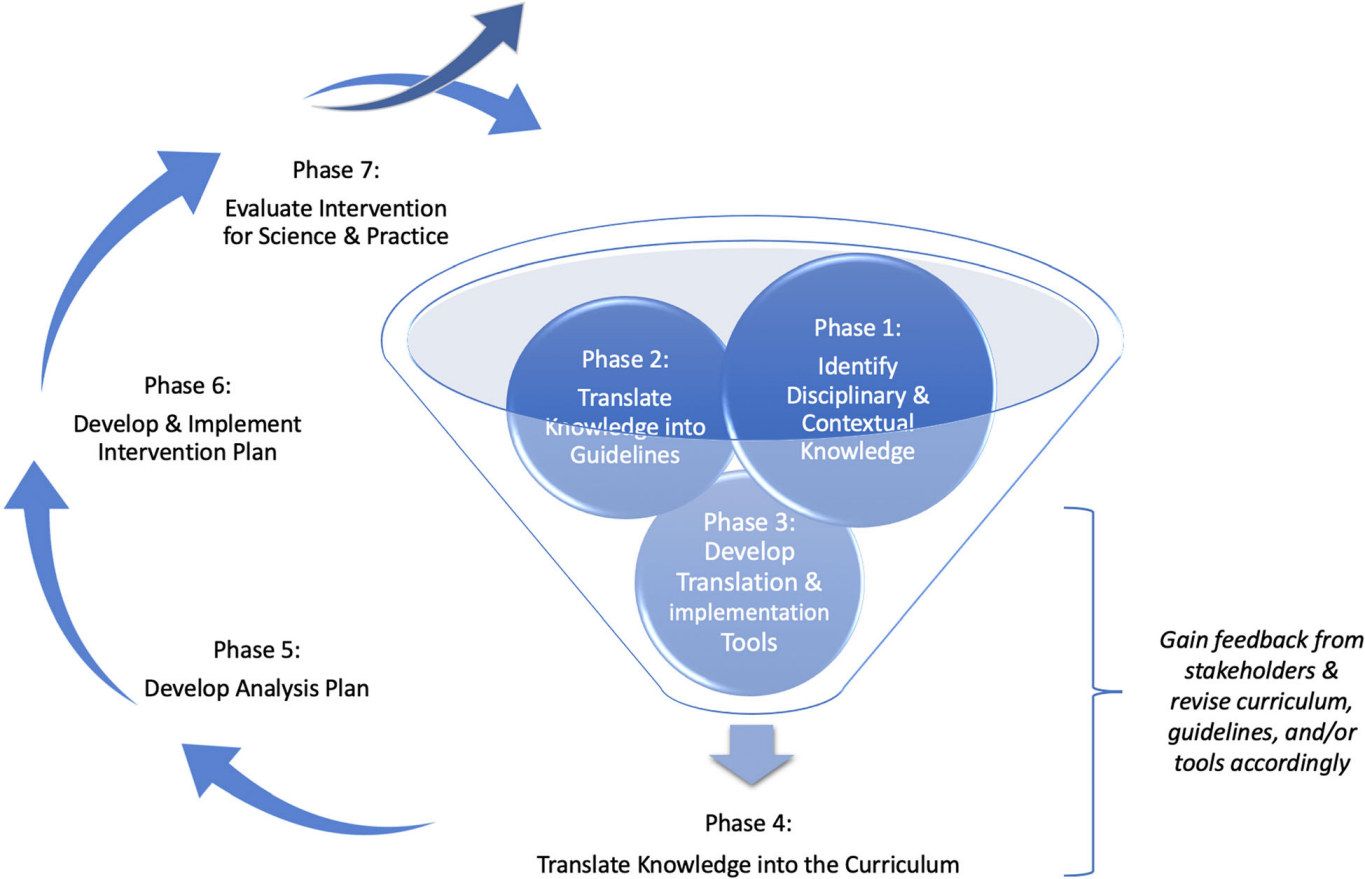
Knowledge Translation Framework

As seen in Fig. 1, the KTF proceeds through seven phases. (1) the identification of relevant disciplinary and contextual knowledge, (2) the synthesis and translation of knowledge into guidelines to support the infusion of knowledge into the curriculum, (3) the development of tools to support curriculum development, implementation, and track the translation process, (4) the iterative development and refinement of the spatially-enhanced curriculum, (5) the creation of an analysis plan to evaluate the impact of the spatial enhancements and other contextual features on learning, (6) the development and implementation of an intervention plan, and (7) the evaluation of the intervention to inform curriculum improvements and scientific knowledge.

The framework is conceptualized as an iterative process, in which knowledge gained from each cycle will inform curriculum improvements or refinements, and expand the scientific knowledge base. Moreover, we present the phases sequentially for illustrative purposes, yet the process is often more complex and dynamic. For example, working on the development of tools in phase 3 may uncover new questions, which will require a team to revisit previous phases and revise their work. Others may find themselves proceeding through phases simultaneously due to a compressed timeline or availability of previously synthesized information.

In this paper, we walk the reader through an illustrative example of how this framework was used to spatially enhance an elementary science curriculum. We provide a broad overview of the goals and activities during each phase and how it guided our translation work. We also provide a description of the tools that we developed to support teachers’ implementation of the curriculum, data systems to track how we infused spatial enhancements into each lesson, and an overview of the analytic plan to determine *how* these spatial enhancements influence learning. This work is part of a large, ongoing curriculum development and intervention project.

### Phase 1: Identify disciplinary and contextual knowledge base

The goal of Phase 1 is to identify the knowledge necessary to spatially enhance the curriculum. We first explored the theoretical rationale for why spatial thinking is related to science outcomes, and then identified what disciplinary and professional knowledge is needed to translate research into educational practice across three main knowledge areas: (a) what scientific knowledge is relevant for translating spatial thinking into a curriculum, (b) what are the needs, interests, and resources in the educational context, and (c) what are the components of the curriculum?

#### Why is spatial thinking related to science outcomes?

**Fig. 2:**
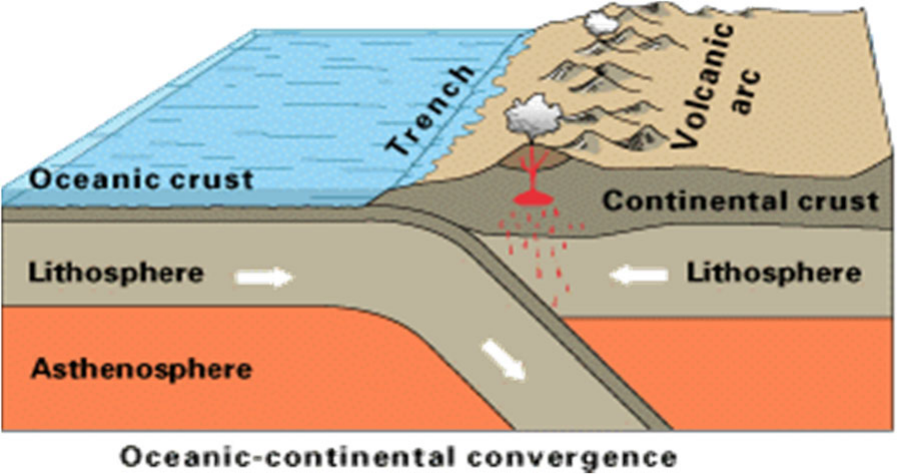
Depiction of tectonic plate movement. https://upload.wikimedia.org/wikipedia/commons/thumb/2/29/Active_Margin.svg/1280px-Active_Margin.svg.png

Understanding scientific phenomena requires the student to: (1) understand space (e.g., location, size, shape, and volume), (2) use tools to represent and interpret space (such as graphs, maps, and diagrams), and (3) reason about and solve spatial problems (such as predicting phases of the moon, learning layers of the earth’s structure, or visualizing three-dimensional (3D) DNA molecules, National Research Council, 2006). An illustration of how scientific learning relies on spatial thinking skills is shown in Fig. 2. The diagram depicts tectonic plate movement and its relations to volcanic eruptions. In order to use this diagram to support learning, a student must first be able to (1) relate the lines in the diagram to 3D properties of the earth, (2) understand that the direction of the arrows indicates the movement of different parts of the lithosphere, and (3) mentally visualize how the movements of these plates influences the volcanic arc and how they would result in new land formation.

Individuals vary dramatically in spatial skills and their skill level is associated with science learning. For example, students who have higher spatial skills learn more from visualizations than students with low spatial skills (Höffler, 2010). As such, a student with weaker spatial skills may be at a disadvantage if the science content relies on spatial thinking without providing support for developing these skills. Initial discomfort felt from being unable to understand or complete tasks or earning poor marks could lead to feelings of failure, which, in turn, could lead to decreased interest and subsequent engagement in STEM subjects and career paths (Covington, 2000; Darnon, Butera, Mugny, Quiamzade, & Hulleman, 2009).

#### What scientific knowledge is relevant for translating spatial thinking into a curriculum?

We synthesized scientific knowledge from multiple fields to develop the spatially-enhanced curriculum, which is briefly outlined below. First, we surveyed the literature on spatial thinking skills and STEM learning to identify spatial enhancements that have been shown to build spatial thinking skills and have been used to support students’ learning in a STEM area. We identified five spatial enhancements that meet these criteria:

- *Visualization Instruction.* Visualizations, such as maps, graphs, and diagrams, are external representations that convey spatial information (e.g., a map depicting the positions of cities across the country or a graph showing sunset time across the year). These representations summarize spatial information that would be difficult to gain from words or direct experience. Visualizations are common in science learning yet challenging for students (Cromley et al., 2013; Newcombe, 2013). Providing instruction on how to interpret spatial patterns in visualizations can support learning (Cromley et al., 2013).
- *Sketching.* A growing body of work has shown that sketching can facilitate science learning (Prain & Tytler, 2012; Tytler, Haslam, Prain, & Hubber, 2009; Van Meter & Garner, 2005; Van Meter, et al., 2006; Van Meter & Firetto, 2013; Waldrip, Prain, & Carolan, 2010), and. in particular. that sketching supports inferential reasoning about spatial properties (Gagnier, Atit, Ormand, & Shipley, 2016; Gobert & Clement, 1999; Gobert, 2000, 2005; Johnson & Reynolds, 2005).
- *Gesture.* A large body of work has shown that gestures – the intentional movement of the arm or head to express an idea or meaning – can support spatial reasoning, aid communication of spatial information (Alibali, 2005), and facilitate student learning in math and science (Crowder, 1996; Goldin-Meadow, 2011; Goldin-Meadow & Alibali, 2013; Liben, Christensen, & Kastens, 2010; Roth, 2000).
- *Spatial comparison.* Analogy facilitates learning through the process of comparison (see Gentner & Smith, 2012; Vendetti, Matlen, Richland, & Bunge, 2015). By virtue of comparing two entities, their commonalities and differences become salient (Gentner & Markman, 1997). Comparison is particularly useful for spatial phenomena to allow learners to notice critical spatial differences between two entities (Matlen, Vosniadou, Jee, & Ptouchkina, 2011).
- *Spatial language.* Spatial language is the use of terms that denote the spatial properties and organization of the world (e.g., on, above, greater than, diagonal). Spatial language can serve to organize spatial information into meaningful units and guide attention to spatial properties and relations (Pruden, Levine, & Huttenlocher, 2011).

Second, we recognized that the implementation of the spatial enhancements into the curriculum needed to be carefully planned to support, and not distract, from the learning experience. If incorporated effectively, the spatial enhancements would support students’ understanding and reasoning about the spatial properties underlying scientific concepts. It would also be important for teachers to scaffold students’ use of the enhancements. Effective scaffolding includes contingent interactions between the teacher and student, with the teacher transferring responsibility for the activity (or parts of the activity) to the student and fading their assistance over time (Van de Pol, Volman, & Beishuizen, 2010).

Drawing on education research, we identified effective instructional scaffolds that could be combined with the spatial enhancements to support learning, including teachers modeling a spatial enhancement to represent a science concept, providing explanations for how the spatial enhancement represents the science concept, asking questions to prompt or guide students’ use of a spatial enhancement, and providing explicit feedback on the use of a spatial enhancement (Oh, 2005; Pea, 2004; Renninger & Granott, 2005; Van de Pol et al., 2010). We developed a plan to incorporate spatial enhancements into each lesson plan, with teachers modeling and explaining spatial enhancements more when introducing new concepts and fading their scaffolds over time. We also expected higher levels of teacher assistance at the beginning of the year and fading over the year, as students become more accustomed to using the techniques. We also identified specific developmental theories and instructional practices that support students’ learning. For instance, developmental theory and research suggest that students actively construct their knowledge through exploration and reflection, and active, high-quality instructional practices should guide the sense-making process in each lesson (e.g., Hamre, Pianta, Mashburn, & Downer, 2007; Koedinger, Corbett, & Perfetti, 2012).

Third, we drew upon key findings in implementation science (e.g., Carroll, Patterson, Wood, Booth, Rick, & Balain, 2007; Metz & Bartley, 2012; O’Donnell, 2008; Yarbrough, Shulha, Hopson, & Caruthers, 2011) to identify common challenges in the implementation of curricula and other programs, including identification of issues that influence the usability and feasibility of the curriculum (e.g., clarity of objectives and instructions, access to available training and resources, leadership support) and potential barriers related to teachers’ foundational knowledge and skills (e.g., spatial thinking skills, understanding of educational standards and effective practices) and implementation (e.g., professional development, need for feedback on their performance). This information was used to inform translation and implementation tools, as well as the structure of lesson plans in subsequent phases.

 Fourth, we drew upon the teacher professional development literature to guide the development of the teacher curriculum training sessions (Darling-Hammond, Hyler, and Gardner 2017; Desimone, 2009; Garet, Porter, Desimone, Birman, Yoon, 2001). This work has identified several core features of effective teacher professional learning programs, which include active participation, collaborative activities, and periods of reflection; content-focus on a specific subject matter (i.e., science vs. general learning principles) and alignment with state standards and local educational goals; sufficient duration over an extended period of time; and ample opportunities to receive feedback on their knowledge and skills from expert coaches.

Together, knowledge from these bodies of research informed the content and delivery mechanisms of the spatially-enhanced curriculum.

#### What are the needs, interests, and resources in the educational content?

As noted earlier, critical barriers to translation arise from scientists or “translators” failing to understand and address the learning needs, resources, and interests of the school context. We propose working closely with educational partners – including teachers, school leaders, curriculum developers, and trainers – throughout the translation process to draw upon their professional knowledge, understanding of the local context, and infrastructure supports to enable effective implementation of the innovation. If a relationship between researchers and a school has already been established, it will be easier to openly discuss learning needs, available resources for use, and other contextual factors at play. If not, this process can take a longer investment period to establish a relationship and build trust. The most successful partnerships between researchers and practitioners are equal, mutually respectful relationships in which all team members recognize, respect, and value the critical expertise that each member brings to the project.

For example, we partnered with a local school district to co-develop a spatially-enhanced third-grade science curriculum in Maryland. The state recently adopted the Next Generation Science Standards (NGSS) and the district planned to develop new lesson plans to align with the standards over the upcoming years. The standards are designed to help children develop a cohesive understanding of science disciplinary core ideas, cross-cutting concepts, and scientific practices through active learning experiences (National Research Council, 2014). To do so, the standard NGSS lesson includes the 5E instructional sequence, which includes five distinct sections designed to build students' knowledge over the course of each lesson: (1) *during Engagement,* students make connections between a scientific phenomenon with what they currently know and can do, (2) *during Exploration*, students work independently or with others to explore their ideas about the phenomenon through hands-on activities, (3) *during Explanation,* students explain their understanding of the concepts and processes they are learning and teachers clarify students’ understanding, (4) *during Elaboration,* students apply their newfound knowledge in new situations and contexts, and (5) *during Evaluation,* students assess their own knowledge, skills, and abilities.

It has been noted that NGSS relies more on spatial thinking than previous standards (e.g., Cromwell, 2014), in that NGSS lessons encourage more hands-on experiences and use of  spatial tools, such as graphs, diagrams, and other visualizations to solve scientific problems; however, the standards provide minimal guidance on how to teach these skills. This presented an opportunity to include more supports for spatial thinking in the third-grade science curriculum. We began outlining the parameters of the spatially-enhanced science curriculum project, including the overarching goals of the project, specific details necessary to develop a curriculum (e.g., amount of class time dedicated to science instruction, flexibility in the school day, resources available in schools, curriculum staff availability and expertise in NGSS, district-level requirements for curriculum evaluation projects), the identification of an interdisciplinary curriculum development team and network of stakeholders that can provide support and feedback (e.g., school leaders, curriculum developers, teachers, evaluation office personnel), teacher demographics and professional development opportunities, and student demographics.

Communication and joint decision-making are paramount to the success of a researcher-practitioner partnership. To ensure open communication, continuous dialogue, and alignment of project goals, we held monthly leadership meetings throughout the project which were attended by district leaders and researchers. The goals of these meetings were to outline and maintain the goals and timeline of the project and address barriers to project success. Through these meetings, our team developed a common language and understanding around NGSS, spatial enhancements, and how the spatial enhancements would be used in the curriculum to build scientific knowledge and skills. During curriculum development, the team met weekly to brainstorm ideas for incorporation of the spatial enhancements into the curriculum and to develop the spatially-enhanced lessons (described in detail in Phase 4).

#### What are the components of the curriculum?

Many translation models do not provide examples of *where* the synthesized knowledge should be incorporated into the curriculum. In some cases, the synthesized knowledge can inform the development of a stand-alone educational experience (e.g., spatial thinking after school club or a daily “spatial play” experience) or the creation of a new curriculum, while others may infuse the synthesized knowledge into an existing curriculum. In our spatially-enhanced science curriculum project, it was clear, from our discussions with school leaders, that there was not enough free time in the school week to dedicate explicitly to spatial thinking training activities, which are the predominant approach in the spatial thinking intervention literature (Lowrie et al., 2017; Miller & Halpern, 2013; Sorby, 2009; Taylor & Hutton, 2013). Thus, we chose to infuse spatial enhancements directly into the district’s existing curriculum. For each lesson, we chose to infuse spatial thinking enhancements into three curriculum components:

- *Pedagogy*. The method by which teachers convey knowledge, with a specific focus on the strategies that they use to facilitate students’ deeper understanding of content. These include guided discovery, elicited student explanations, and cues to prompt recall of prior knowledge (e.g., Alfieri et al., 2011; Alibali, 2005; Fisher, Hirsh-Pasek, Newcombe, & Golinkoff, 2011; Hmelo-Silver, Duncan, & Chinn, 2007; Klahr & Nigam, 2004; Van de Pol et al., 2010; Vygotsky, 1978; Wood, Bruner, & Ross, 1976).
- *Teaching and Learning Materials*. The physical resources that teachers use to support the presentation of the curriculum (e.g., visual representations used in a presentation slide deck such as charts or graphics) and the physical resources that students utilize during a lesson (e.g., worksheets, manipulatives, and technologies).
- *Support Materials*. Additional classroom resources that guide learning (e.g., textbooks, online learning laboratories, classroom posters).

We found it helpful to develop a Theory of Change to represent how the integration of this knowledge can lead to anticipated outcomes. We also recommend developing a Logic Model to represent how this information translates into key program components and activities of the curriculum (e.g., inputs, outputs) and operationalized outcomes (short-term, intermediate-term, long-term).

### Phase 2: Translate Knowledge into Guidelines

Phase 2 represents the first translation step, in which knowledge from disparate fields (described in Phase 1) is synthesized and translated into guidelines to support its infusion into the curriculum. Drawing from our knowledge of the extant literatures, we were able to identify five spatial enhancements that could be incorporated into lessons, scaffolding strategies that teachers could use to support students’ effective use of the enhancements, and general guiding principles to support the creation of effective, high-quality lessons and teacher professional development trainings. We also identified three areas in each lesson that we could incorporate the spatial enhancements and scaffolding strategies – including pedagogy, teaching and learning materials, and other supports. Lastly, we defined the parameters of the educational context to understand the structure, content, and frequency of the spatially-enhanced lessons; resources available in the classroom settings; potential translation and implementation barriers; and learning needs of the students.

There were also several areas that the literature could not directly address. For instance, the extant literature provided little practical guidance on how often a spatial enhancement should be used in the lesson to impact spatial thinking skills, if one enhancement or a combination of enhancements were more effective for specific lesson content (e.g., gesture is best for lessons about weather, but not inherited traits?), or how to effectively scaffold the use of the enhancements for different learners.

Through an initial series of discussions, our team collectively came up with a set of guiding questions to support the synthesis and systematic application of the spatial enhancements based on the available evidence. When developing the lessons (described in Phase 4), each team member would address the following questions independently, and, during our weekly meetings, we would discuss our answers and refine the lesson accordingly based on team consensus:

- What spatial enhancement(s) best lend itself to support the learning of the specific science content and structure of the lesson? Why?
- Does the inclusion of the spatial enhancement(s) facilitate conceptual understanding or accurate science or engineering practice use?
- Can the lesson, with all chosen spatial enhancements, be completed in the allotted lesson time frame?
- Will the proposed spatial enhancement(s) lead to differences in time spent in the spatially-enhanced lesson compared to the business-as-usual lesson?
- Are we using a range of spatial enhancements in the lesson? Across lessons?
- Were the spatial enhancements used frequently throughout the lesson or were they used at one point, and referenced subsequently?
- Did the lesson employ active, high-quality instructional practice?
- Did the lesson clearly articulate teacher’s scaffolding strategies to support the students’ use of the spatial enhancements?
- Does the lesson plan accurately utilize the NGSS 5E lesson plan structure, address the relevant NGSS standard(s), and lesson objectives?
- Are there any specific considerations for diverse learners?

**Fig. 3:**
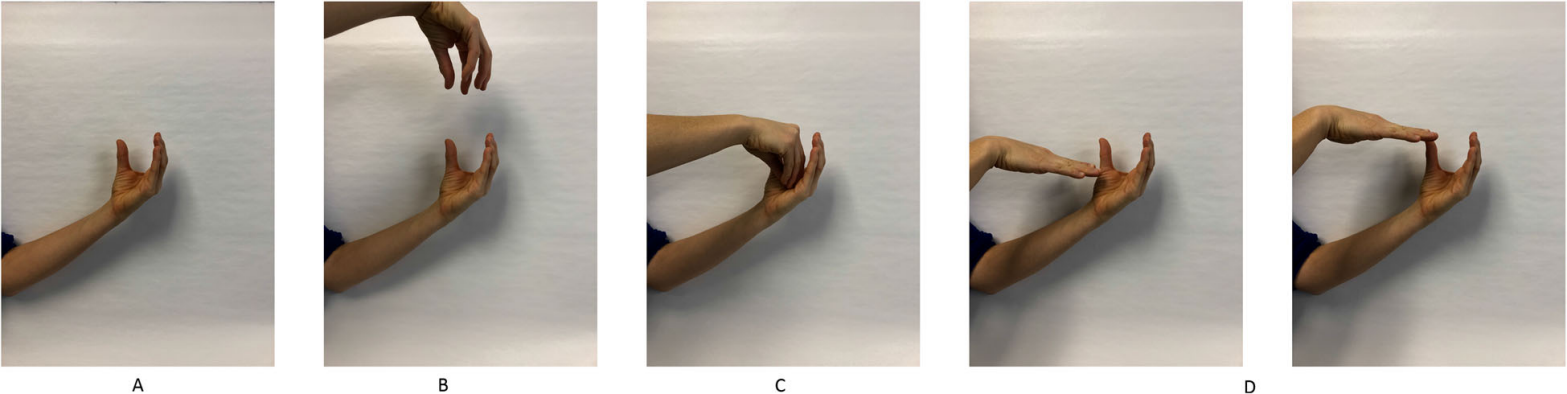
A temporal sequence illustration of how gesture is used to enhance a teacher's explanation of the function of a rain gauge. Picture **a**: The hand represents an empty rain gauge with an opening on top. Picture **b**: The top hand lowers while the fingers move repeatedly back and forth to represent rain falling downward. Picture **c**: The top hand lowers into the bottom hand to represent the rain falling into the the rain gauge. Picture **d**: The top hand represents the low water level in the rain gauge when it first begins to rain. Picture **e**: The top hand moves upward, representing the rising water level in the rain gauge as it continues to rain over time

To provide an illustrative example, consider a lesson aimed at building students’ understanding of how weather tools are used to provide data on various types of weather (e.g., a rain gauge provides data on the amount of precipitation in a region). The original, or business-as-usual, lesson included a slide presentation to show students pictures of a rain gauge followed by a verbal description that a rain gauge is used to collect water. Our curriculum development team, which included experts in spatial thinking, developmental science, and science curriculum development, chose to include instruction in visualizations, spatial language, and gesture in teachers’ pedagogy to demonstrate how a rain gauge works. A teacher would show a picture of a rain gauge, use gestures (shown below in Fig. 3) and spatial language to describe the shape of the rain gauge, demonstrate how it collects rain, and how the water level increases over time as it continues to rain. Throughout the lesson, the teacher and students return to this example. The team proceeded to address each question listed above to maximize the use of the spatial enhancements to support science learning, while at the same time, maintain feasibility for classroom practice.

### Phase 3: Develop Translation and Implementation Tools

The goal of Phase 3 is to develop tools to support the systematic translation of the research into the curriculum and set up structures to track and monitor this process. With this goal in mind, we developed three types of tools to support implementation at multiple levels: (1) a *Curriculum development tool* which supports the systematic implementation of the spatial enhancements into the curriculum, (2) *Curriculum implementation tools* which support the implementation of the curriculum in the classroom (i.e., tools to support the teacher), and (3) *Curriculum refinement tools* which were used to refine and improve the usability and feasibility of curriculum.

#### Curriculum development tool

**Fig. 4:**
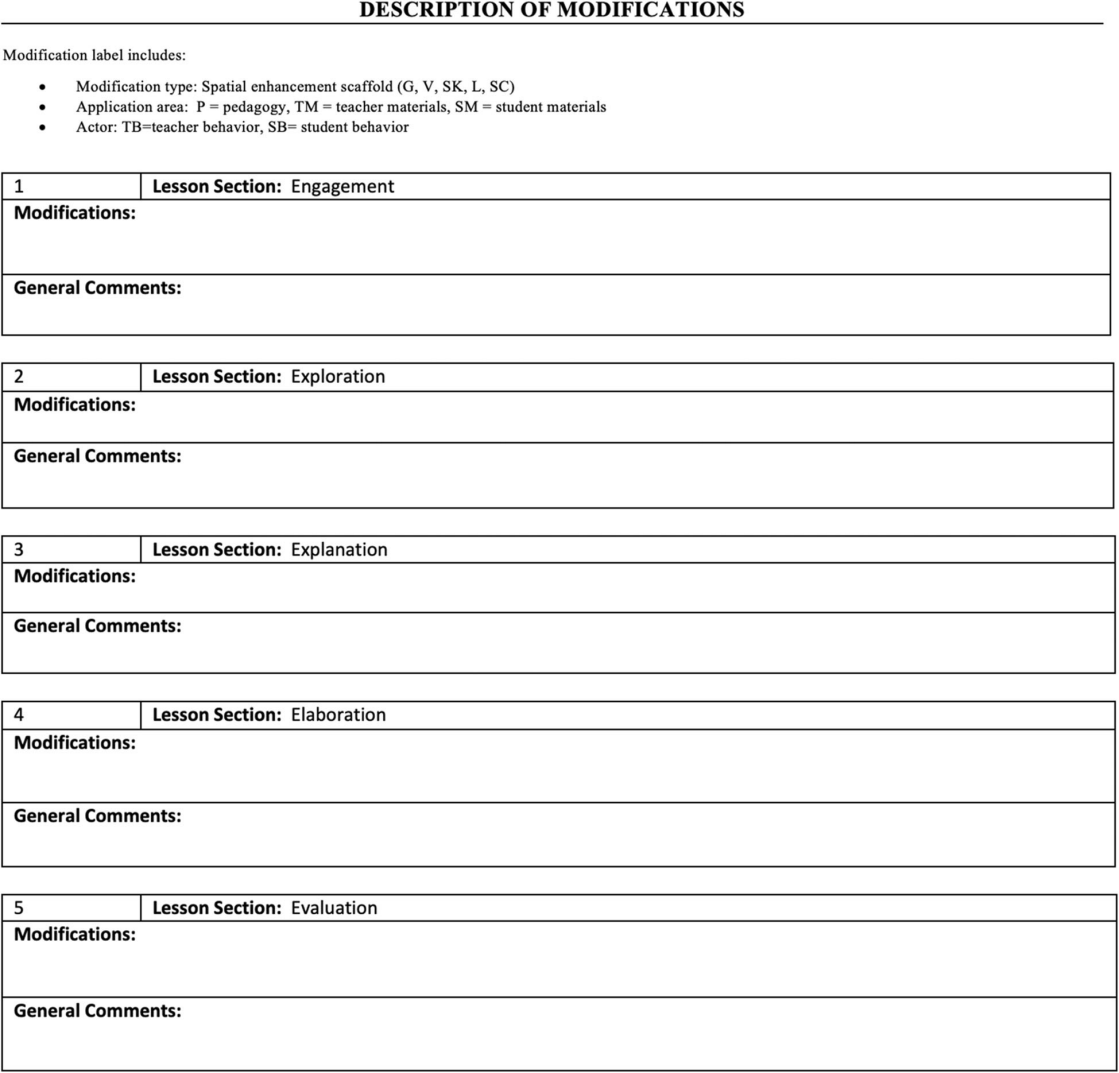
Spatial Enhancement Crosswalk Template

To facilitate the systematic incorporation of the spatial enhancements into the curriculum, according to our outlined guiding questions described under Phase 2, we created the Spatial Enhancement Crosswalk Template to record and track the addition of spatial enhancements into the curriculum. As seen in Fig. 4, the template parallels the 5E NGSS lesson structure. For each 5E section, we reviewed the business-as-usual lesson description and described how we would modify this section of the lesson to incorporate spatial enhancements. The modifications were described in sufficient detail so that the team members could fully conceptualize the modification and a curriculum developer could write it into the curriculum.

We used a three-tier coding system to categorize each spatial enhancement and track the extent to which we utilized the enhancements within and across lessons. For each modification, we coded the type of spatial enhancement used (modification type), the location in which the spatial enhancement was infused into the three curriculum components (application area), and the person who implemented the enhancement, either the teacher or the student (actor).

To create this coding system, we identified five codes to represent each of our five spatial enhancements, G = Gesture, V = Visualization instructions, L = Spatial language, SC = Spatial comparison, and SK = Sketching. Second, we created three application area codes to document where in the curriculum the spatial enhancement would take place, P = pedagogy (what the teacher says and does during the lesson), TM = teacher materials (i.e., PowerPoints, Google Slides, or other materials the teacher uses to teach with), and SM = student materials (i.e., worksheets, activity instructions, or any other materials that students engaged with during the lesson). Finally, we created two actor codes to identify who in the lesson would implement the spatial enhancement, TB = teacher behavior, and SB = student behavior. It was important to recognize that the teacher, the student, or both may perform the spatial enhancements during the lesson. For instance, the teacher may model the use of the enhancement while providing instruction or can prompt students to use the enhancement.

**Fig. 5:**
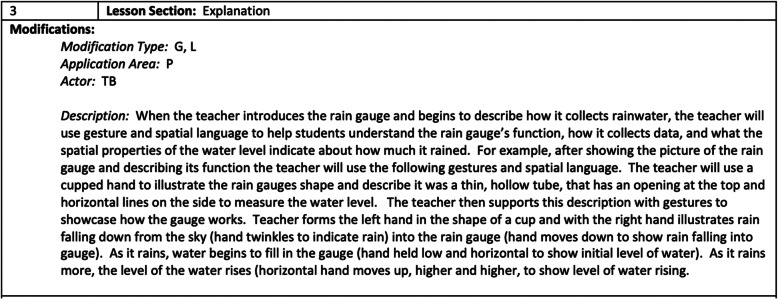
Completed section of the Spatial Enhancement Crosswalk Template

Figure 5 illustrates the crosswalk template completed for a sample explanation section, in which the teacher uses instruction in visualizations, gesture, and spatial language to support students’ understanding of the function of a rain gauge introduced earlier in Phase 2. As can be seen in the figure, the crosswalk contains codes for the type of modifications used (i.e., G = gesture, L = language), location of modifications (i.e., P = teacher pedagogy), and actor (i.e., TB = teacher behavior). The description contains sufficient detail regarding the language and gestures used to allow the modifications to be written into the curriculum.

**Fig. 6:**
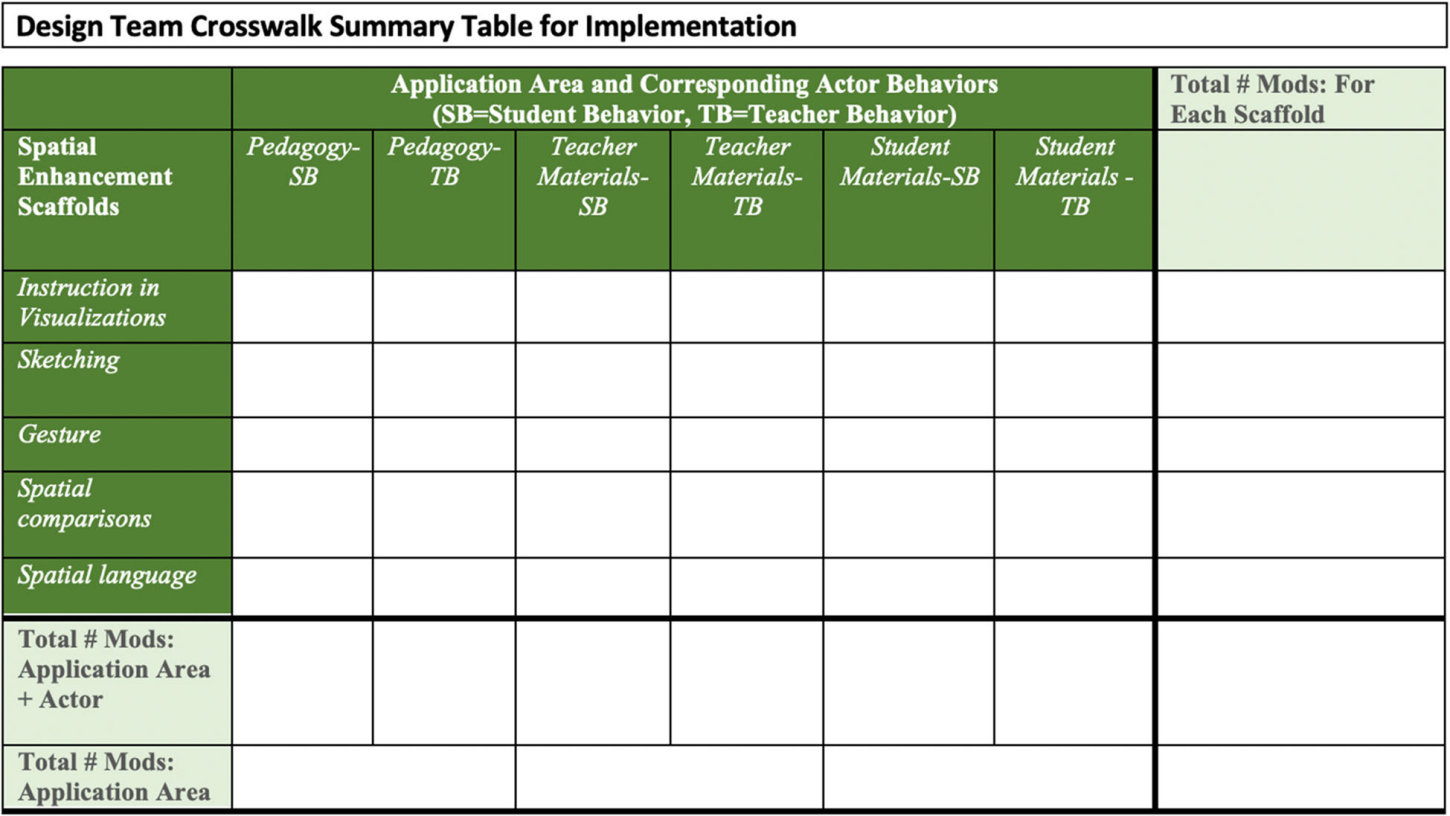
Design Team Crosswalk Summary Table

After identifying and coding the modifications in a lesson, we would record the total number of spatial enhancements in the Design Team Crosswalk Summary Table, shown in Fig. 6, which served two purposes. First, we could easily track the total number of spatial enhancements made in each lesson, broken down by type of enhancement (instruction in visualizations, sketching, gesture, spatial comparison, spatial language), application area (e.g., pedagogy), and actor. Second, we could easily visualize the distribution of spatial enhancements within and across lessons, which would help illuminate uneven application of the spatial enhancements and adjust, as necessary.

#### Curriculum implementation tools

Working with curriculum development experts, we discussed potential limits to teacher knowledge that would serve as a barrier to successful curriculum implementation and identified lesson supports to help the teachers. To support teachers as they implemented the spatially-enhanced curriculum in their classroom, we created three types of instructional supports to be used across lessons: (1) worked-example lesson plan, (2) spatial enhancement icons, and (3) a “spatial word bank” poster.

*Worked example lesson plans.* If our curriculum simply noted that teachers should utilize a list of spatial enhancements in each lesson to support students’ understanding of a science concept or practice, the instruction could be interpreted and implemented in multiple ways. We felt that it was critical to provide teachers with a framework to see how each spatial enhancement is used to build students’ conceptual understanding of science and address each lesson objective. In the same way that reviewing a worked example can be helpful in learning to solve math problems (Atkinson, Derry, Renkl, & Wortham, 2000), we wanted to show teachershow the enhancements unfold in the lesson. To this end, we created worked example lesson plans that showed how spatial enhancements were used to support students' understanding of the spatial features underlying the scientific concepts and practices in each of the 5E sections of a lesson plan, utilizing. corresponding materials (e.g., teacher slide deck presentation, student worksheets) and lesson supports.

We included specific language and descriptions of all sketches, comparisons, gestures, and instructions (see Fig. 5 and 8b for details). As the lessons became quite long, we also included a one- to two-page “at a glance” summary of the spatial enhancements used in each section of the lesson.

**Fig. 7:**
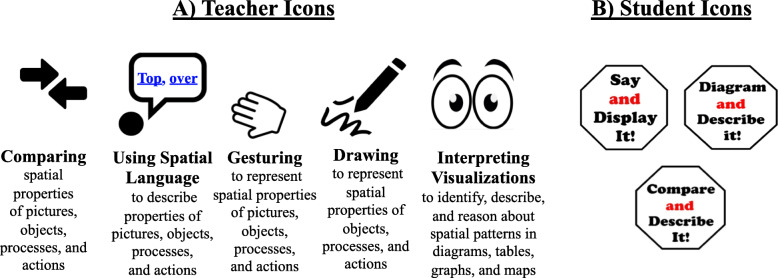
Icons denoting spatial enhancements in the curriculum

*Spatial enhancement icons for teachers and students.* To support teachers’ use of the the spatial enhancements, we incorporated five “teacher icons” into the lesson plan as a visual indicator of the spatial enhancements *they* should be using in each section of the lesson. Examples of the teacher icons are shown in Fig. 7a.

**Fig. 8:**
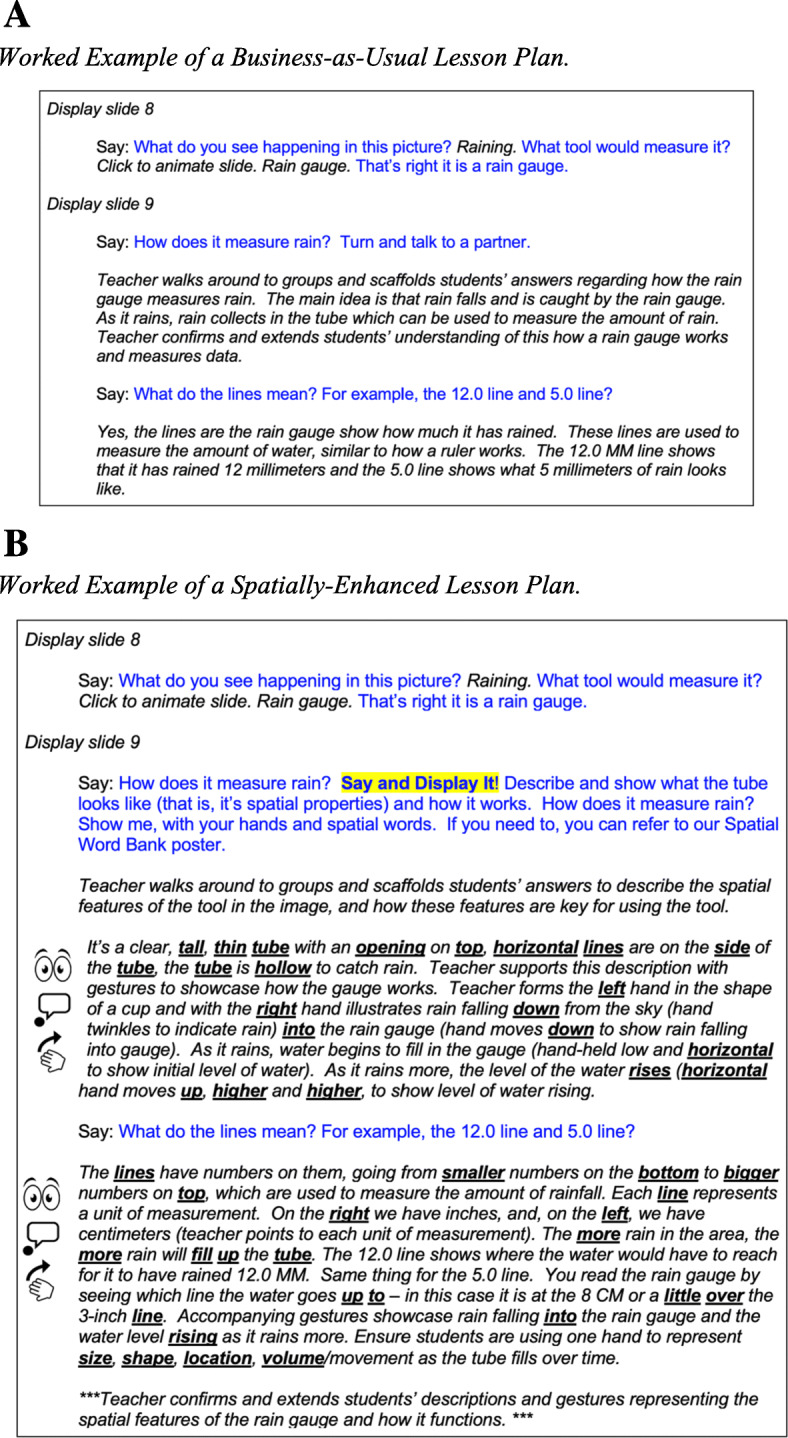
**a** Worked example of a “business-as-usual” lesson plan. **b** Worked example of a spatially-enhanced lesson plan

We also created eight common student activities that would be utilized throughout the year during science lessons. The common activities combined various spatial enhancements in fun and engaging ways. For example, in a “Say and Display It!” activity , students use spatial language and gesture to describe and show their observations, phenomena, or predictions of science concepts or practices (see Fig. 8b for a lesson example ). A list of common student activities and their descriptions can be found in the Appendix. We created “student icons” for each common student activity, which appeared in  the slide deck presentations and student materials (e.g., worksheets) as a visual reminder to students of what they should say and do . An example of the three student icons appears in Fig. 7b. While both teachers and students utilize all spatial enhancements in the curriculum, we created different visual icons and prompts to help distinguish expectations for teachers and students.

*Spatial word bank poster.* We created a classroom spatial word bank poster that teachers and students could reference throughout the year to describe spatial properties, phenomena, and observations. The poster lists words divided into eight broad categories of spatial language outlined by Cannon, Levine, and Huttenlocher (2007): spatial dimensions, shapes, locations and directions, orientations and transformations, continuous amount, deictics, spatial features and properties, and patterns.

#### **Curriculum refinement tools**

A critical step in program development is to solicit feedback from various stakeholders who will use the program, product, or tools. For this project, we solicited feedback from stakeholders to get input on (1) the usability and feasibility of the curriculum, (2) the usability of the tools and process for developing the spatially-enhanced curriculum, and (3) the extent to which the spatial enhancements are used consistently throughout the lesson to support content knowledge development. Together, the data from these three tools inform the refinement of the spatially-enhanced curriculum.

*Lesson usability and feasibility survey.* We developed a 16-item survey to assess teachers’ perceptions of the usability and feasibility of each  spatially-enhanced lesson. The survey included statements which teachers rated on a 5-point scale from not at all to extremely. In this context, usability refers to alignment of the lesson content with the learning objectives and the clarity of instructions for implementation of the curriculum (e.g., Are the instructions sufficiently clear for you to implement the spatial enhancements effectively?) Feasibility refers to the degree to which the lesson, as currently written, is likely to have an impact in the classroom (e.g., what is the likelihood of you using all the spatial enhancements while teaching, as described in the lesson plan?). Teachers were also asked to provide qualitative suggestions of ways to improve the lesson. The data from this survey guided future refinement and improvement efforts.

*Usability of curriculum development tool.* Following the initial production of five spatially-enhanced lessons, we solicited feedback from all members of the curriculum development team regarding their use of the Spatial Enhancement Crosswalk Template. Based on the reported ease of use and synthesis of spatial enhancements into the curriculum, we made several modifications to the template to improve the usability of our curriculum development processes and tools.

*Extent of spatial enhancement use throughout lesson survey.* We created a 10-item survey to solicit expert feedback on the extent to which the lesson encouraged both students and teachers to use each of the five spatial enhancements consistently throughout the lesson in support of students’ understanding of the spatial features underlying a science concept or practice. Experts in spatial thinking and science learning rated each lesson using a 5-point scale from not at all (spatial enhancement was not used by either the teacher or the student to support students’ understanding) to extremely (the spatial enhancement was used consistently throughout the entire lesson by teachers/students to support students’ understanding ).

### Phase 4: Translate Knowledge into Curriculum

Phase 4 represents the second translational step in which knowledge is infused into the curriculum. The goal of Phase 4 is to leverage the synthesized knowledge, translation guidelines, and tools to *systematically* translate research into the curriculum. This translation is an iterative process, which includes: (1) developing the spatially-enhanced curriculum, (2) soliciting usability and feasibility feedback from experts (as described in Phase 3), and (3) revising the curriculum, tools, and guidelines based on feedback.

#### Developing the spatially-enhanced curriculum

 Utilizing the overarching guidelines and tools for curriculum development, implementation, and revision, our curriculum development team began to develop the spatially-enhanced curriculum. Our interdisciplinary team of experts first completed the Spatial Enhancement Crosswalk Templates independently. The team met to discuss their crosswalk templates, compare ideas, and choose which modifications would best support the lesson objective (see Phase 2 rubric for detailed description of selection guidelines). Next, our curriculum developer wrote the worked-example lesson that incorporated the chosen spatial enhancements and recorded the results in the Design Team Crosswalk Summary Table (Fig. 6).

To illustrate this process, Fig. 8 presents the business-as-usual (Fig. 8a) and the spatially-enhanced lesson plan (Fig. 8b) for the rain gauge example. As can be seen in the figure, there is blue text and black italic text. This formatting was consistent throughout our lessons. Blue text describes what the teacher should say, in an ideal situation, and provides teachers with an understanding of how the lesson progresses and key concepts to convey using the spatial enhancements. The black italic text provides guidance for the teacher on how to scaffold using spatial enhancements. For example, what language to use during instruction (spatial language is bolded and underlined) and how to gesture. As can be seen by comparing across the two examples, the spatially-enhanced lesson plan contains more detailed information for teachers on how to bolster student understanding of the rain gauge through the use of the spatial enhancements in their pedagogy.

Also evident in the spatially-enhanced lesson plan (Fig. 8b) is a student activity highlighted in yellow. For example, in this Say and Display It! activity, students use spatial language and gesture to describe the spatial features of the rain gauge and how the rain gauge is used to collect rain data. A list of common student activities and their descriptions can be found in the Appendix. Note, the student activity icon for the Say and Display It! activity (see Fig. 6b) does not appear in the teachers’ lesson plan. As described in Phase 3, student icons appeared in all classroom resources that students would see.

After each spatially-enhanced lesson was written, we tracked and monitored the incorporation of all spatial enhancements within and across these lessons using the template shown in Fig. 6 and described in Phase 3. By monitoring the use and application of the spatial enhancements, we ensured that the curriculum development team applied the spatial enhancements in accordance with the guidelines established during Phase 2. Monitoring and tracking the application of spatial enhancements during translation into the curriculum positioned our team to analyze the structure of the spatially-enhanced curriculum (described in Phase 5, evaluation).

#### Soliciting usability and feasibility feedback

Following the production of the spatially-enhanced lessons, we solicited feedback from experts using the tools described in Phase 3: (1) the usability and feasibility of the curriculum and (2) the spatial enhancement use throughout the lesson. 

First, we asked a group of eight third-grade teachers to review and rate each lesson’s perceived usability and feasibility, as well as provide suggestions on ways to improve the lessons. During a series of focus groups, we presented the data to the teachers and solicited in-depth feedback on lessons that were rated moderate to low on usability and/or feasibility. Second, we asked experts in spatial thinking and science learning to rate the extent to which the spatial enhancements were used consistently throughout the lesson.

#### Revising the curriculum based on feedback

During the revision phase, the curriculum development team incorporated suggested revisions on the methods for improving the usability and feasibility of the curriculum and revised lessons to ensure that the spatial enhancements were used consistently throughout the lessons to build content knowledge. The team also ensured that any changes to the enhancements still aligned with the guidelines outlined in Phase 2.

Based on our experiences and lessons learned throughout this project, we recommend taking an iterative approach to lesson development, if possible. We suggest developing a small number of lessons and soliciting feedback from stakeholders. This will maximize the potential impact of the curriculum and streamline the development process by systematically addressing challenges and barriers as they arise in the development process (rather than after full curriculum is developed).

### Phase 5: Develop Analysis Plan

Many intervention studies focus predominantly on examining the impact of an educational intervention on students learning, while little focus is given to examining the characteristics of the curriculum itself. To this end, the goal of Phase 5 is to create an analysis plan to examine the extent to which the synthesized knowledge has been translated into the curriculum and how such features of the curriculum influence learning outcomes in future intervention work.

To explore the characteristics of the curriculum, we used the tracking system (see Fig. 6) data to examine how spatial enhancements were systematically implemented within and across lessons to address questions such as: Which spatial enhancements were consistently used across all lessons? Were certain spatial enhancements more prevalent in certain topical areas? Moreover, content analysis of this nature paves the way for a more detailed summative evaluation in the future. Our ongoing intervention work will examine the differential impact of the spatially-enhanced science curriculum and the business-as-usual curriculum on students’ spatial thinking skills, academic achievement, and interest in science. Our approach, outlined in this paper, should also provide us with the means to answer questions such as: which spatial enhancements seemed more effective, and under what contexts in the curriculum (e.g., location in the curriculum, actor) and for whom? Are there dosage effects, such that lessons with higher numbers of spatial enhancements build more knowledge than lessons with less enhancements? A well-planned analytic approach will be an integral component in bridging the research-practice gap, providing information on how, when, and to what extent using these types of spatial enhancements facilitate learning, which, up until this point, has not been possible.

### Phases 6 and 7: Implementation and Evaluation

The goal of this paper was to provide a detailed framework to support the initial translational steps to infuse research on spatial thinking, cognitive, developmental, and educational sciences, and implementation science into a science curriculum. The first five phases outlined in the KTF are, of course, just the beginning; the ultimate goal of such translation is to bring researchers and practitioners together to improve educational outcomes for teachers and students through the implementation and evaluation of research-informed educational programs.

Now, we turn briefly to the remaining phases in the KTF. The goal of Phase 6 is to develop and implement the intervention plan. While the development of the research design is often completed alongside the analysis plan, special care must be taken in planning the *implementation process*, given that poorly planned, low-quality implementation structures can dramatically affect the impact of an intervention (Moir, 2018). This phase begins with confirming that all curricular elements remain in place in the classroom (e.g., materials are available in the classrooms, the science instruction schedule remains the same) and establishing a timeline and protocol for data collection. It also includes establishing appropriate infrastructure supports for teachers implementing the curriculum, with organized access to instructional materials (e.g., lesson plans, slide decks, computers, and projector), clear protocols for implementing the curriculum, and access to training assistance.

We highly recommend that readers carefully consider the different implementation drivers at play in their educational contexts and include relevant measures in their data collection protocol (e.g., Bauer, Damschroder, Hagedorn, Smith, & Kilbourne, 2015; Lyon, 2020; Moir, 2018). Implementation drivers are key components of capacity and infrastructure that influence a program’s success (National Implementation Research Network, 2013). For example, some may wish to include assessments of teacher competencies (e.g., science, spatial skill, and teaching knowledge, implementation fidelity), leadership support (leadership style, school climate or culture), and other factors that can impact curriculum implementation (e.g., professional development quality, dosage of professional development training, perceived usability and feasibility of lessons). It would be helpful to actively monitor the data to better understand how the elements of the larger system can interact with the intervention during implementation.

Phase 7 focuses on evaluating the outcomes of the intervention. During this phase, team members follow the data analysis plan from Phase 5 and, if applicable, explore how implementation factors moderate or mediate the effects of the intervention on learning. Team members share this knowledge with relevant stakeholders in the educational context (e.g., teachers, school leaders, curriculum developers) as well as with the broader scientific community. The knowledge gained from the evaluation phase should yield important insights into how the team members, teachers, and leaders can improve the curriculum as well as contribute to a burgeoning scientific knowledge base that has practical, real-world implications.

## Discussion

Many reading this paper will question the feasibility of the approach outlined in the KTF, noting that this approach may be difficult and resource-intensive. This is true, particularly for those who venture out to do this work for the first time. This process requires a dedicated interdisciplinary team with expertise in relevant sciences (e.g., education, cognitive, child development, and implementation sciences) and topical domains (e.g., spatial thinking, instructional quality, instruction and scaffolding), knowledge translation, professional expertise (e.g., curriculum development, science standards), and who have deep familiarity with the educational context. The sobering truth is that bridging research and practice is incredibly challenging. This reality is why the two often remain siloed or result in the implementation of underdeveloped, superficial programs, with minimal impact. The difficulty of this problem was one reason why our team invested much time and resources into the development of protocols, processes, templates, and other tools. The tools and approaches offered in this paper are a first step in streamlining this process and creating structures that make this process more feasible for future researchers and practitioners.

We hope that this framework will serve as a mechanism to simplify the translation process of incorporating research into educational experiences. In our illustrative example, we used the framework to translate spatial thinking research and other relevant knowledge into a spatially-enhanced curriculum and associated products. Curriculum development is but one of many potential translation products. After school programs and other learning experiences (e.g., camp programs, museum exhibits, library programs) as well as teacher professional training programs could be produced utilizing the KTF. Future translational science work should explore the viability and impact of a variety of products to bridge the research-practice gap.

## Conclusion

Effectively translating research on spatial thinking into educational practice requires a framework to support systematic, rigorous, and careful translation that preserves the scientific integrity of the research base and meets the needs of the educational context. In this paper, we offer a use-inspired, integrative framework that draws upon research from cognitive, developmental, educational, and implementation sciences to guide the initial infusion of spatial thinking into a curricular intervention. The KTF builds upon planned action and translational science frameworks (Graham et al., 2006) by (a) unpacking the translation process and (b) offering practical guidance on how a team may synthesize scientific and contextual knowledge, infuse it into a curriculum, and evaluate its impact in ways that will yield scientific understanding *and* practical knowledge.

The virtues of this framework are multifold, benefiting both the advancement of scientific knowledge and educational practice in several ways. First, the framework, to our knowledge, is one of the first of its kind to delineate a use-inspired approach in the generation, synthesis, translation, and application of knowledge for our field and, more specifically, for spatial thinking. Second, it illuminates critical elements in the translation process that are often overlooked in the spatial thinking intervention work and the larger field, including who should be included in the translation process, what knowledge should be identified and synthesized, and how and where the knowledge should be translated into educational experiences.

Third, it highlights the importance of utilizing an interdisciplinary team of scientists and practitioners to produce and translate synthesized knowledge into the real-world context. Arguably, involving key stakeholders in the process, from production to application, will increase the likelihood of effective translation and uptake. Fourth, the utilization of this framework should play an integral role in bridging the translation gap. The framework provides a detailed, yet flexible, roadmap for researchers and educators on how to translate spatial thinking knowledge into educational curricula and other learning experiences. Finally, it should yield new scientific knowledge regarding what spatial enhancement approaches are most effective in supporting science learning and under what conditions. This should contribute to our basic understanding of spatial thinking and its role in science learning and achievement, in ways that were not possible without such a framework.

## Data Availability

Not applicable

## Appendix

Descriptions of the eight common student activities:

*Compare and Describe It!* (Comparison and Language): Students compare two or more properties within a single image (e.g., bars in a single graph) or two or more images. Images can include pictures, graphs, or designs to identify their similarities or differences. Students may be asked to provide a verbal or written description highlighting the similarities or differences.

*Describe It!* (Language): Students use spatial language to describe scientific phenomena and practices. They use spatial words during verbal descriptions or adding annotations, labels, and/or descriptors to images, graphs, diagrams, and other visualizations.

*Design, Compare, and Describe It!* (Comparison, Sketching, and Language): Students sketch a design to address a problem. They compare their designs to other students’ designs or, if they made revisions to their initial designs, their final design. During this comparison, they will use spatial words, labels, and/or descriptors to highlight spatial features and how the designs are similar/different.

*Diagram and Describe It!* (Sketching, Language, Visualizations): Students sketch a diagram and describe its components. This includes (1) arrows to represent spatial properties, such as movement, directionality, cause and effect, passage of time, or sequence of phases, and (2) spatial words, labels, and/or descriptors to the diagram.

*Graph It!* (Sketching): Students create a graph (e.g., bar, line) from data that they collect during experiments, from data that the teacher provides to them, or from class-generated data.

*Observe, Draw, and Describe It!* (Sketching and Language): Students draw their observations and label the components of the important features or elements of the observations. Students describe the properties of their observations using spatial words, labels, and/or descriptors.

*Predict, Draw, and Describe It!* (Sketching and Language): Students (1) draw a representation of their own prediction that is a result of forces, processes, events, or design choices based on their new learning and evidence, and (2) add spatial words, labels, and/or descriptors to their drawings.

*Say and Display It!* (Gesture and Language): Students use spatial language and gesture to describe and show their observations, phenomena, predictions of scientific content.
